# Compare Breast Cancer Screening, Diagnosis, and Treatment Between Medicare Patients With and Without ADRD

**DOI:** 10.1093/geroni/igaa057.478

**Published:** 2020-12-16

**Authors:** Xingran Weng, Chan Shen, Monali Vasekar, Sachin Gupta, Li Wang

**Affiliations:** 1 Penn State College of Medicine, Hershey, Pennsylvania, United States; 2 Tower Health Medical Group, Wyomissing, Pennsylvania, United States

## Abstract

Background: Incidence of both breast cancer and Alzheimer’s disease and related dementias (ADRD) increases with advancing age. Little research has delineated breast cancer screening, diagnosis, and treatment among women with ADRD. Method: Surveillance, Epidemiology, and End Results (SEER)-Medicare linked data were used. Female breast cancer patients diagnosed between 2005-2015 were identified. Chi-square tests were conducted to compare the characteristics of two groups with and without ADRD. Multiple logistic regression models were estimated to explain the diagnosis and treatment differences. Results: A total of 44,112 female Medicare beneficiaries age 65 or older were identified. Patients with ADRD (17.5%) were less likely to receive breast cancer screening (42.8% vs. 46.6% for all data years combined, p<0.0001), more likely to be diagnosed with breast cancer after death by autopsy or death certificate (8.1% vs 2.0%, p<0.0001). Among those who are diagnosed before death, patients with ADRD were more likely to be diagnosed with breast cancer at age 75 and older (84.8% vs. 15.2%, p<0.0001). After adjusting for age, race, poverty level, marital status, cancer stage at diagnosis, cancer screening history, wellness visit history, comorbidity, and rural/urban residence, logistic regressions suggest that patients with ADRD were less likely to receive surgery (AOR=0.48, 95%CI: 0.45-0.52), radiation (AOR=0.41, 95%CI: 0.39-0.44), or chemotherapy (AOR=0.38, 95%CI: 0.35-0.41). Conclusion: Breast cancer screening was less utilized and breast cancer was diagnosed at an older age in patients with ADRD than those without. Treatments (surgery, radiation, and chemotherapy) were given less frequently to patients with ADRD.

